# Factors influencing exclusive breastfeeding practice among under-six months infants in Ethiopia

**DOI:** 10.1186/s12884-022-04955-x

**Published:** 2022-08-08

**Authors:** Gizachew Gobebo Mekebo, Alemayehu Siffir Argawu, Habte Tadesse Likassa, Wondimu Ayele, Senahara Korsa Wake, Dechasa Bedada, Belema Hailu, Temesgen Senbeto, Ketema Bedane, Kebede Lulu, Sagni Daraje, Reta Lemesa, Gudeta Aga, Endale Alemayehu, Bizunesh Kefale, Terefa Bechera, Getachew Tadesse, Agassa Galdassa, Jiregna Olani, Geribe Hemba, Girma Teferi, Abebe Argaw, Tariku Irana, Tsigereda Tilahun, Gezahagn Diriba

**Affiliations:** 1 Department of Statistics, Ambo University, Ambo, Ethiopia; 2 Department of Statistics, Addis Ababa University, Addis Ababa, Ethiopia; 3 Department of Biostatistics and Epidemiology, Addis Ababa University, Addis Ababa, Ethiopia; 4 Department of Midwifery, Wolkite University, Wolkite, Ethiopia

**Keywords:** Exclusive breastfeeding practice, Factors influencing, Under-six month infants, Ethiopia

## Abstract

**Background:**

World Health Organization recommends exclusive breastfeeding (EBF) for the first 6 months of life. EBF has sustainable long-term health benefits for both infants and mothers. Despite its benefits, the practice of EBF in Ethiopia is lower than the internationally recommended one. This study aimed at identifying factors influencing EBF practice among under-6 month infants in Ethiopia.

**Methods:**

This study used data drawn from the 2019 Ethiopian Mini Demographic and Health Survey (2019 EMDHS) data. A multivariable logistic regression model was employed to investigate factors significantly associated with EBF practice among under-6 month infants in Ethiopia. An adjusted odds ratio with 95% confidence interval was used to measure the association of factors with EBF practice.

**Results:**

A total of 566 infants under the age of 6 months were included in the study. The prevalence of exclusive breastfeeding practice was 83% (95% CI: 79.70–86%). Urban residences (AOR: 0.40, 95% CI: 0.22–0.73), mothers having secondary education (AOR: 1.54, 95% CI: 1.29–1.84) and higher education (AOR: 3.18, 95% CI: 0.68–15.02), mothers having ANC visits of 1 to 3 times (AOR: 1.52, 95% CI: 1.24–1.88) and ANC visits of 4 and more times (AOR: 4.27, 95% CI: 1.06–17.25), family size of more than 5 (AOR: 0.45, 95% CI: 0.26–0.88), caesarean births (AOR: 0.63, 95% CI: 0.42–0.95), and deliveries at health facilities (AOR: 2.51, 95% CI: 1.12–5.63) were factors significantly associated with EBF practice among under-6 month infants.

**Conclusion:**

In this study, EBF practice among under-6 month infants was significantly associated with place of residence, maternal educational level, ANC visits, family size, mode of delivery, and place of delivery. Therefore, encouraging ANC visit and promotion of institutional (health facility) delivery are recommended. Furthermore, special attention has to be given to mothers with no or less education to make them better aware of the EBF and its benefits to enhance exclusive breastfeeding practice.

## Background

Exclusive breastfeeding (EBF) is defined as the infant receiving only breast milk, no other liquids or solids even water, except for oral rehydration solution, or drops/syrups of vitamins, minerals or medicines [1]. EBF is the safest and healthiest feeding option of infants for the first 6 months of their lives. The World Health Organization (WHO) recommends all mothers to feed their infants solely with breast milk for the first 6 months of their infants’ life [2]. EBF is recommended because breast milk is uncontaminated and contains all the nutrients necessary for an infant to attain optimal growth and health [3]. EBF is a basis for survival and health of an infant since it provides necessary and irreplaceable nutrition required for growth and development of the infant. EBF also serves as the first immunization of a child by protecting from diseases like diarrhoeal disease, respiratory infections, and life-threatening illnesses [4].

The WHO also recommends EBF for the first 6 months of life, followed by continued breastfeeding with appropriate complementary foods until 2 years and beyond [5]. Breastfeeding maintains the optimal health status of the infant like providing nutrients, immunity, and improved developmental outcomes, and it reduces the risk of developing asthma, diabetes mellitus, and obesity [6]. The breastfeeding practices can be affected by various factors like cultural, socio-economic, and individual factors related to the infant and mother [7]. The breastfeeding decline is associated with women involvement in the workforce, lack of knowledge on the benefits of the practice [8].

EBF has sustainable long-term health benefits to both infants and mothers. It has benefits to infants in that it reduces neonatal mortality, risk of childhood obesity, and enhances growth and cognitive development, and it also has benefit to mothers in that it reduces the risk of breast and ovarian cancers [5, 9, 10]. The EBF is associated with demographic, socioeconomic, maternal, socio-cultural, and psychosocial support factors [11].

According to the WHO report, 41% of infants under the age of 6 months are breastfed exclusively [12]. In developing countries, the prevalence of exclusive breastfeeding among infants under the age of 6 months was 39% [13]. In Africa, about 37% of infants under six months of age were exclusively breastfed in 2017 [14]. The WHO set a target to increase the rate of exclusive breastfeeding in the first six months globally to at least 50% by 2025 [3], and to at least 70% by 2030 [12]. Increasing breastfeeding could prevent 823,000 deaths in children under age five and 20,000 deaths from breast cancer annually [15].

In 2006, the Government of Ethiopia developed the second phase of the National Nutrition Program (NNP II, 2016–2020) that, among other targets, targeted promoting and encouraging mothers to exclusively breastfeed their child for the first 6 months without any additional fluids or foods [16]. In spite of the fact that Ministry of Health of Ethiopia has been doing all its best to enhance and implement exclusive breastfeeding practices for infants under the age of 6 months, no remarkable improvement has been seen in the country. Nationally in Ethiopia, exclusive breastfeeding among infants under the age of 6 months has increased by only 1% from 2016 (58%) [17] to 2019 (59%) [18].

Earlier studies in various countries show EBF practice is associated with various socio-economic, demographic, obstetric and health care factors related to child and mother such as size of child at birth, sex of child, age of child, birth order, preceding birth interval, place of delivery, mode of delivery, mother’s current age, mother’s current marital status, mother’s age at first birth, mother’s level of educational, desire for pregnancy, mother’s occupation, mother’s religion, place of residence, geographical region, household wealth index, antenatal care (ANC), postnatal care (PNC) [19–48]. Other influencing factors of EBF practice include parity, family size, smoking, professional counselling on breastfeeding, infant feeding counselling, initiation of breastfeeding, and mother’s knowledge about EBF [22, 23, 26, 27, 29, 31, 33–35, 40, 42, 46, 49–51].

Globally, infant optimal breastfeeding practice is among the most effective intervention areas that were identified to achieve the SDG of reducing child mortality. Despite its benefits, the practice of EBF in Ethiopia is lower than the internationally recommended one. Identifying influential factors associated with exclusive breastfeeding is very crucial to inform policymakers and program implementers to implement appropriate interventions to reduce infants’ mortalities and morbidities due to the lack of necessary nutrients, and to reduce their mothers’ risk of developing diseases. This study will also have a contribution to the promotion of exclusive breastfeeding practices. Various studies have been conducted on EBF practice in Ethiopia but no study has been conducted using the most recent national representative data, 2019 Ethiopia Mini Demographic and Health Survey (2019 EMDHS) data. Therefore, this study aimed to identify factors influencing exclusive breastfeeding practice among under-6 months infants in Ethiopia based on the 2019 EMDHS data.

## Methods

### Data source and study design

The 2019 EMDHS data were used for the study. The 2019 EMDHS is the second EMDHS implemented in Ethiopia. The 2019 EMDHS is a national representative cross-sectional survey data which has been implemented by the Ethiopian Public Health Institute, in partnership with the Central Statistical Agency and the Federal Ministry of Health, under the overall guidance of the Technical Working Group from March 21 to June 28, 2019. The 2019 EMDHS generates data for measuring the progress of the health sector goals set under the Growth and Transformation Plan (GTP), which is closely aligned to the Sustainable Development Goals (SDG). The survey interviewed 8,855 women of reproductive age (age 15–49) from a nationally representative sample of 8,663 households. Detailed information was collected on respondents’ background characteristics, fertility determinants, marriage, awareness and use of family planning methods, child feeding practices, nutritional status of children, childhood mortality, and height and weight of children aged 0–59 months [18].

### Study variables

#### Outcome variable

The outcome variable is EBF practice which is a dichotomous variable categorized as 1 if a mother did not feed the baby anything else except syrup and medicine apart from breast milk and 0 otherwise.

#### Independent variables

The independent variables included in this study were Demographic and socioeconomic factors (sex of child (categorized into male and female), birth order of child (categorized into 1, 2–3, 4+), initiation of breastfeeding (categorized into ≤ 1 h of birth and > 1 h of birth), mother’s current age ( categorized into 15–24 years, 25–34 years, and 35 years and above), maternal level of education (categorized into no education, primary, secondary, and higher), mother’s current marital status (categorized into never in union, currently in union, and widowed/separated), mother’s religion (categorized into protestant, orthodox, Muslim, and others), place of residence (categorized into urban and rural), region (categorized into Dire Dawa, Tigray, Afar, Amhara, Oromia, Somalia, Benshangul Gummuz, SNNPR, Gambela, Harari, Addis Ababa), household wealth index (categorized into poor, middle, rich), household family size (categorized into < = 5, more than 5), parity (categorized into 1–2, 3–4, 5 and more )) and Obstetric and health care related factors (Antenatal visits (categorized into 0, 1–3, and 4+), place of delivery (categorized into home and health facility), Caesarean delivery (categorized into no and yes), postnatal check within 2 months (categorized into yes and no), Counselling on breastfeeding by health provider during first 2 days of a birth (categorized into yes and no)).

### Statistical data analysis

Data analysis was done using SPSS version 26. Descriptive summary measures like percentage, frequency, mean, and standard deviation were used to describe the background characteristics of the infants and mothers. First, bivariate analysis was conducted to select the candidate variables for multivariable analysis. The multivariable analysis was conducted considering those independent variables with p-values of less than 0.05 in bivariate analysis. In the multivariable analysis, the adjusted odds ratio with 95% confidence interval and p-value less than 0.05 were used to identify the significant factors influencing EBF among under 6 month infants.

The presence of multicollinearity among independent variables was checked using Variance inflation factor (VIF). It was found that VIF values of all variables were less than 10, which implies that there was no multicollinearity among independent variables. And, the model goodness of fit was checked using Hosmer and Lemeshow Test. Hosmer and Lemeshow test result showed p-value of 0.290, which implies that the model is a good fit.

## Results

### Prevalence of EBF

In this study, the prevalence of EBF practice among under-6 months infants in Ethiopia was 83% (95% CI: 79.70, 86%).

### Background characteristics of infants and mothers

In this study, a total of 566 infants under the age of 6 months were included. Slightly more than half (52.30%) of them were females and the remaining 47.70% of them were males. About two-fifth (44.17%) of the infants had birth order of fourth and above. Majority (82.16%) of the infants were initiated breastfeeding within 1 h of birth (Table 1).

**Table 1:** Background characteristics of infants and mothers in Ethiopia, EMDHS 2019 (*n* = 566)

Variables	Categories	Frequency	Percent
Sex of child	Female	296	52.30
	Male	270	47.70
Birth order	1st	131	23.15
	2nd -3nd	185	32.69
	4th and above	250	44.17
Initiation of breastfeeding	≤ 1 h	465	82.16
	> 1 h	101	17.85
Age of mother in years	15–24	199	35.16
	25–34	283	50
	35+	84	14.84
Maternal level of educational	No education	278	49.12
	Primary	193	34.10
	Secondary	64	11.31
	Higher	31	5.48
Marital status	Never in union	8	1.41
	Currently in union	543	95.94
	Widowed/separated	15	2.65
Religion	Orthodox	157	27.74
	Protestant	102	18.02
	Muslim	302	53.36
	Others	5	0.88
Place of residence	Rural	446	78.80
	Urban	120	21.20
Region	Dire Dawa	42	7.42
	Tigray	56	9.90
	Afar	66	11.66
	Amhara	44	7.77
	Oromia	73	12.90
	Somalia	60	10.60
	Benshangul Gummuz	51	9.01
	SNNPR	58	10.25
	Gambela	49	8.66
	Harari	43	7.60
	Addis Ababa	24	4.24
Household wealth index	Poor	294	51.94
	Middle	72	12.72
	Rich	200	35.34
Family size	<=5	253	44.70
	> 5	313	55.30
Parity	1–2	233	41.17
	3–4	153	27.03
	5+	180	31.80
Antenatal care visits	0	151	26.68
	1–3	185	32.69
	4+	230	40.63
PNC check within 2 months	No	512	90.46
	Yes	54	9.54
Caesarean delivery	No	529	93.46
	Yes	37	6.54
Place of delivery	Home	255	45.05
	Health facility	311	54.95
Counseling on breastfeeding by health provider during first 2 days of a birth	No	368	65.02
	Yes	198	34.98

The mean age of the mothers was 27 years with a standard deviation of 6.30, and exactly half (50%) of the women were in the age group of 25–34 years. Regarding educational level, nearly half (49.12%) of them had no education and only 31(5.48%) of them had higher than secondary education. Regarding marital status, more than nine-tenth (95.94%) of mothers were in union at the time of survey. About 157 (27.74%), 102(18.02%), 302 (53.36%), and 5 (0.88%) them were respectively Orthodox, protestants, Muslims, and other religion followers. Nearly two-fifth (78.80%) of mothers were living in rural areas, and 73(12.90%) were from Oromia region. About half (51.94%) of the mothers were with poor wealth indices, and 313 (55.30%) of them had family size of more than five. About 233 (41.17%), 153 (27.03%), and 180 (31.80%) of mothers respectively had total ever born 1 to 2 children, 3 to 4 children, and 5 and more children (Table 1).

Nearly three-fourth (73.33%) of mothers had ANC visits for their recent pregnancy. Nearly three-fourth (73.32%) of the mothers attended at least one ANC visit. Of the mothers who attended ANC visits, about two- fifth (40.63%) of them attended four and more visits. Only one-tenth (9.54%) of mothers received a postnatal check within 2 months. More than nine-tenth (93.46%) of mothers gave birth via Caesarean delivery. About 311 (54.95%) of mothers gave births at health facilities, and 198 (34.98%) of mothers were counselled on breastfeeding by health care providers during the first two days of their births (Table 1).

### Bivariate analysis

Table 2 shows that EBF practice varies by the characteristics of infants and mothers. There was only quiet slight difference in percentage of EBF practice among female infants and male infants. Highest percentage of EBF practice (85.41%) was also observed among the infants with births order of second and third. Regarding initiation of breastfeeding, higher EBF practice (88.17%) was among infants who were initiated within 1 h of birth.

**Table 2:** Bivariate analysis of EBF practice by characteristics of infants and mothers in Ethiopia, EMDHS 2019 (*n* = 566)

Variables	Categories	EBF		*P*-value	COR(95% CI)
		**Yes (n, %)**	**No (n, %)**		
Sex of child	Female	246(83.11)	50(16.89)		1
	Male	224(82.96)	46(17.07)	0.36	0.10(0.64–1.536)
Birth order	1st	104(79.39)	27(20.61)		1
	2nd -3nd	158(85.41)	27(14.59)	0.27	2.12(0.93–4.82)
	4th and above	208(83.20)	42(16.80)	0.03^a^	4.53(1.64–12.52)
Initiation of breastfeeding	≤ 1 h	410(88.17)	55(11.83)		1
	> 1 h	60(59.41)	41(40.59)	0.51	1.87(0.31–11.40 )
Age of mother in years	15–24	168(84.42)	31(15.58)		1
	25–34	233(82.33)	50(17.67)	0.64	1.18(0.60–2.32)
	35+	69(82.14)	15(17.86)	0.97	1.01(0.54–1.91 )
Maternal educational level	No education	221(79.50)	57(20.50)		1
	Primary	169(87.56)	24(12.44)	0.04^a^	1.29(1.24–1.34)
	Secondary	55(85.94)	9(14.06)	0.03^a^	1.50(1.44–1.553 )
	Higher	25(80.65)	6(19.35)	0.03^a^	2.60(0.55–12.27)
Marital status	Never in union	7(87.50)	1(12.50)		1
	Currently in union	450(82.87)	93(17.13)	0.96	1.08(0.08–14.08)
	Widowed/separated	13(86.67)	2(13.33)	0.70	0.74(0.17–3.35)
Religion	Orthodox	142(90.45)	15(9.55)		1
	Protestant	94(92.16)	8(7.84)	0.45	2.37 (0.25–22.57)
	Muslim	230(76.16)	72(23.84)	0.36	2.94 (0.29–29.51)
	Others	4(80)	1(20)	0.84	0.80 (0.09–7.26)
Place of residence	Rural	369(82.74)	77(17.26)		1
	Urban	101(84.17)	19(15.83)	0.01^a^	0.37(0.20–0.68)
Region	Dire Dawa	32(76.19)	10(23.81)		1
	Tigray	53(94.64)	3(5.36)	0.27	0.46(0.11–1.86)
	Afar	42(63.64)	24(36.36)	0.28	2.52(047-13.52)
	Amhara	42(95.45)	2(4.55)	0.04^a^	0.25(0.07–0.93)
	Oromia	67(91.78)	6(8.22)	0.28	3.00(0.47–19.35)
	Somalia	29(48.33)	31(51.67)	0.03^a^	0.60(0.50–0.71)
	Benshangul Gummuz	51(100)	0(0)	0.04^a^	0.13(0.04–0.50)
	SNNPR	54(93.10)	4(6.90)	0.91	1.51(0.96–2.35)
	Gambela	45(91.84)	4(8.16)	0.42	1.33(0.28–6.26)
	Harari	34(79.07)	9(20.93)	0.56	1.61(0.33–7.83)
	Addis Ababa	21(87.50)	3 (12.50)	0.03^a^	1.85(1.18–2.90)
Household wealth index	Poor	235(79.93)	59(20.07)		1
	Middle	63(87.50)	9(12.50)	0.28	0.65(0.40–1.06)
	Rich	172(86)	28(14)	0.75	1.14(0.51–2.55)
Family size	<=5	215(84.98)	38(15.02)		1
	> 5	255(81.47)	58(18.53)	0.00^a^	0.46(0.25–0.83)
Parity	1–2	189(81.12)	44(18.88)		1
	3–4	134(87.58)	19(12.42)	0.89	0.96(0.59–1.59)
	5+	147(81.67)	33(18.33)	0.34	1.58(0.86–2.92)
Antenatal visits	0	111(73.51)	40(26.49)		1
	1–3	160(86.49)	25(13.51)	0.01^a^	1.35(1.15–1.55)
	4+	199(86.52)	31(13.48)	0.00^a^	3.83(1.68–8.75)
PNC check within 2 months	No	425(83.01)	87(16.99)		1
	Yes	45(83.33)	9(16.67)	0.95	0.98(0.46–2.07)
Caesarean delivery	No	443(83.74)	86(16.26)		1
	Yes	27(72.97)	10(27.03)	0.02^a^	0.60(0.58–0.73)
Place of delivery	Home	195(76.47)	60(23.53)		1
	Health facility	275(88.42)	36(11.58)	0.04^a^	2.43(1.06–5.54)
Counseling on breastfeeding by health provider during first 2 days of a birth	No	297(80.71)	71(19.29)		1
	Yes	173(87.37)	25(12.63)	0.02^a^	2.20 (1.42–3.38)

*COR *Crude odds ratio, *CI *Confidence interval, 1: Reference group, ^a^ Statistically significant

Concerning maternal age, maternal education, and religion, highest percentage of EBF practice was observed among infants belonging to younger age mothers. The percentage of EBF practice was highest (84.42%) among of the infants belonging to mothers aged 15–24 years. Highest percentage of EBF practice (87.56%) was also observed among infants whose mothers attended primary education followed by those whose mothers attended secondary education (85.94%). It was also the highest (92.16%) among the infants born of protestant mothers (Table 2).

With regard to region, place of residence, household wealth index, and parity, the percentage of EBF practice was lowest in the Somali region (48.33%) followed by Affar region (63.64%). The percentage of EBF practice was higher (84.17%) among urban infants than the rural ones. Similarly, it was higher (84.98%) among the infants from family size of less than or equals five. The percentage of EBF practice was highest (87.50%) among the children from families of middle wealth index. It was also highest (87.58%) among infants born of mothers whose total number of children ever born was 3 to 4 (Table 2).

Regarding ANC visits and PNC visits, a higher percentage of EBF practice (86.51%) was observed among mothers who had ANC visits than those who had no ANC visits, but it was not varied with the number of times of visits made. It was also shown that EBF practice was not varied with PNC check within 2 months of the birth. A higher percentage of EBF practice (87.37%) was reported among the infants born to mothers counselled on breastfeeding by health care providers during the first two days of their births. The percentage of EBF practice was higher (83.74%) among infants born vaginally than those born by caesarean. Similarly, it was higher (88.42%) among the infants born at health facilities than those born at home (Table 2).

### Factors associated with EBF practice among under-6 month infants in Ethiopia

The results of multivariate analysis showed that place of residence, maternal educational level, ANC visit, family size, mode of delivery, and place of delivery were factors significantly influencing EBF practice among under-6 month infants. The odds of EBF practice among urban mothers was 0.40 (AOR: 0.40, 95% CI: 0.22–0.73) times lower than those living in rural areas. Mothers with secondary education (AOR: 1.54, 95% CI: 1.29–1.84) and higher education (AOR: 3.18, 95% CI: 0.68–15.02) were more likely to practice exclusively breastfeeding to their babies compared to mothers who had no formal education. Similarly, mothers who had ANC visits of 1 to 3 times (AOR: 1.52, 95% CI: 1.24–1.88) and 4 and more times (AOR: 4.27, 95% CI: 1.06–17.25) were more likely to practice exclusively breastfeeding to their babies compared to those who had no ANC visits at all. Babies with a family size of more than 5 (AOR: 0.45, 95% CI: 0.26–0.88) were less likely to be practiced exclusive breastfeeding than those with a family size of 5 and less. Similarly, babies born by caesarean (AOR: 0.63, 95% CI: 0.42–0.95) were less likely to be exclusively breastfed than those born vaginally. Moreover, babies born at the health facilities (AOR: 2.51, 95% CI: 1.12–5.63) were more likely to be practiced exclusively breastfeeding compared to those born at home (Table 3).

**Table 3:** Factors associated with EBF practice among under-6 months infants in Ethiopia from multivariable logistic regression analysis

Variables	Categories	COR (95% CI)	AOR (95% CI)	*P*-value (AOR)
Birth order	1	1		
	2–3	2.12(0.93–4.82)	2.03(0.74–5.63)	0.42
	4+	4.52(1.64–12.52)	3.82(0.93–15.67)	0.18
Maternal level of educational	No education	1		
	Primary	1.29(1.24–1.34)	1.53(0.41–5.66)	0.42
	Secondary	1.50(1.44–1.55 )	1.54(1.29–1.84)	0.04^a^
	Higher	2.60(0.55–12.27)	3.18(0.68–15.02)	0.02^a^
Place of residence	Rural	1		
	Urban	0.37(0.20–0.68)	0.40(0.22–0.73)	0.01^a^
Region	Dire Dawa	1		
	Tigray	0.46(0.11–1.86)	0.45(0.09–2.20 )	0.33
	Afar	2.52(047- 13.52)	1.90(0.31–11.51)	0.49
	Amhara	0.25(0.07–0.93)	0.42(0.13–1.39)	0.15
	Oromia	3.00(0.47–19.35)	3.29(0.43–24.99 )	0.25
	Somalia	0.595(0.50–0.71)	0.65(0.36–1.18)	0.80
	Benshangul Gummuz	0.13(0.04–0.50)	0.22(0.04–1.09)	0.19
	SNNPR	1.51(0.96–2.35)	1.48(0.85–2.56)	0.10
	Gambela	1.33(0.28–6.26)	1.49(0.26–8.51)	0.66
	Harari	1.62(0.33–7.83)	1.55(0.26–9.34)	0.63
	Addis Ababa	1.85(1.18–2.90)	1.87(0.40–8.83)	0.24
Family size	<=5	1	1	
	> 5	0.46(0.25–0.83)	0.48(0.26–0.88)	0.00^a^
Antenatal care visits	0	1	1	
	1–3	1.35(1.15–1.55)	1.52(1.24–1.88)	0.00^a^
	4+	3.83(1.68–8.75)	4.27(1.06–17.25)	0.01^a^
Caesarean delivery	No	1	1	
	Yes	0.60(0.58–0.73)	0.63(0.42–0.95)	0.03^a^
Place of delivery	Home	1	1	
	Health facility	2.43(1.06–5.54)	2.51(1.12–5.63)	0.02^a^
Counselling on breastfeeding by health provider during first 2 days of a birth	No	1	1	
	Yes	2.20 (1.42–3.38)	2.07(0.90–4.75)	0.09

*COR* Crude odds ratio, *AOR *Crude odds ratio, *CI *Confidence interval, 1: Reference group, ^a^Statistically significant

## Discussion

This study attempted to identify factors influencing exclusive breastfeeding practice among under-6 month infants in Ethiopia. In the study, the prevalence of EBF practice among under-6 month infants was 83% with (95% CI: 79.70, 86%). The prevalence of EBF practice in this study is comparable with prior studies done in Ethiopia ( Dubti town (81.1%) [50], Ambo woreda, (82.2%) [51]) and Northern Ghana (84.3%) [52]. It is higher than the findings of the studies done in Ethiopia (Sheka Zone (76%) (27), Offa district(78%) [43], rural Ethiopia (77.5%) [53], Debre Berhan District (68.6%) [21], Azezo District (79%) [54]), Malawi (71.3%) [55], Zimbabwe (36%) [44], Ghana (71%) [24], India (48.5%) [23], Mexico (28%) [32] and China (29.5%) [29], but lower than the finding of study in Ethiopia (rural SNNPR and Tigray regions (88.0%) [56]. This discrepancy might be due to differences in study period, study design, age distribution of infants, socio-economic status, socio-cultural factors, and health service utilization across the study areas.

In our study, the multivariable logistic regression analysis revealed that place of residence, maternal educational level, ANC visit, family size, mode of delivery, and place of delivery were factors significantly associated with EBF practice among under-6 month infants in Ethiopia.

This study showed that place of residence was significantly associated with EBF practice. The result is comparable with the prior studies from Ethiopia ( Debre Berhan District, and Sheka Zone [21, 26]), Saudi Arabia [57], Cambodia [42]. This might be due to that urban women are mostly engaged in paid work out of their homes, either in permanent or temporary jobs, which makes them spend some/much of their time away from their children. But, this finding disagrees with finding of study from Indonesia [45] which found that women residing in urban areas were more likely to practice EBF than those residing in rural areas.

This study also showed that maternal level of education was significantly associated with EBF practice. The EBF practice improves with increased maternal educational level; more educated mothers were more likely to practice EBF to their infants than mothers with no and little education. This result is consistent with finding of the studies in Ethiopia (Sheka Zone, Offa district, and Azezo district [26, 43, 54]) and Myanmar [58] which revealed that maternal education had positive effect on EBF practice. This result is also supported by studies conducted in Ethiopia (Mecha District [46]) and Indonesia [45] which revealed that more educated mothers were more likely to practice exclusive breastfeeding. The possible explanation could be that mothers with higher level of education.

can be more aware of benefits of EBF by reading written messages from various sources and easily understanding what they are counselled than mothers with no or less education. Mothers who had ANC visit in their recent pregnancy were more likely to practice EBF than those who had no ANC visit at all. This finding is consistent with the finding of prior studies in Ethiopia in (Azezo district, Jimma Town, West shoa zone, and Bahir Dar city [39, 54, 59–61]), Egypt [62], Malawi [55], Myanmar [58] and India [23, 63]. This might be due to the fact that mothers during their ANC visits can get awareness about exclusive breastfeeding practice and its benefits by the health professionals and that might make them encouraged to exclusively breastfeed their infants.

In addition, this study showed that family size was significantly associated with EBF practice. Mothers with larger family size were less likely to practice EBF to their infants than those with smaller family size. This finding agrees with the finding of prior study in Ethiopia (Dilla Zuria District [64]), India [63]. The possible explanation could be that mothers may be busier in handling members of the families in larger family size than those with smaller family size, which might make mothers in larger family size lose attention to breastfeed their infants. But, it disagrees with finding of study in Ghana [24] which revealed that mothers with larger family size were more likely to practice exclusive breastfeeding than those with smaller family size.

Furthermore, our study revealed that mode of delivery was significant factor associated with EBF practice. Mothers who gave birth by caesarean were less likely to practice EBF than those who gave birth vaginally. This finding is consistent with the findings of the prior studies in Ethiopia (Hawassa, and Bahir Dar city [41, 65]), Saudi Arabia [48, 57], Bangladesh [37]. The possible justification could be that caesarean delivery which may result in some pain may make mothers refrain from practicing exclusively breastfeeding compared to mothers delivering vaginally. Additionally, place of delivery was significant factor associated with exclusively breastfeeding practice. Mothers who gave birth at health facilities were more likely to practice EBF than those gave birth at home. This finding is in agreement with the findings of earlier studies in Ethiopia (Sheka Zone, Hawassa, Azezo district, SNNP and Tigray regions, Bahir Dar city, and Gozamin district [26, 39, 41, 54, 56, 65, 66]), Malawi [55], Cambodia [42], and Myanmar [58]. The possible justification could be that when mothers deliver their births at healthy facilities, the health professionals can counsel them to feed their babies exclusively breast milk for the first 6 months [67].

### Strength of the study

The study used the national representative survey data.

### Limitations of the study

The main limitation of the study was the inability to include some of factors of EBF practice like preceding birth interval [64, 68] (due to high missing values in the dataset), size of child at birth [28] (due to unavailability on the dataset), mother’s occupation [29, 54, 66] (due to unavailability on the dataset) and maternal knowledge about importance of EBF [32, 59, 69] (due to unavailability on the dataset), which could have association with EBF were not included in the study. The other limitation was that the survey used 24 h recall method for measuring EBF which might be source of recall or measurement bias. In addition, because of the cross-sectional nature of the data used for the study, it was not possible to establish causal relationship between factors and exclusive breastfeeding practice.

## Conclusion

In the study, we found that that place of residence, maternal educational level, ANC visit, family size, mode of delivery, and place of delivery were significantly associated factors with EBF practice among under-6 month infants in Ethiopia. Therefore, encouraging ANC visit and promotion of institutional (health facility) delivery are recommended. Furthermore, special attention has to be given to mothers with no or less education to make them better aware of the EBF and its benefits to enhance exclusive breastfeeding practice.

## Data Availability

The dataset used for the current study was drawn from 2019 EMDHS which is publically available on https://dhsprogram.com/data/available-datasets.cfm, and the dataset used for the final analysis during the current study can be obtained from the corresponding author on reasonable request.

## Abbreviations

DHS: Demographic and Health Survey
EBF: Exclusive breastfeeding
EMDHS: Ethiopia Mini Demographic and Health Survey
SDGs: Sustainable Development Goals
SNNP: Southern Nations, Nationalities and Peoples
SNNPR: Southern Nations, Nationalities and Peoples Region
UNICEF: United Nations Children’s Fund
VIF: Variance Inflation Factor
WHO: World Health Organization
